# Development of a PCR‐RFLP assay to identify *Drosophila melanogaster* among field‐collected larvae

**DOI:** 10.1002/ece3.4453

**Published:** 2018-09-25

**Authors:** Vincent Raquin, Hélène Henri, Marine Vallat, François Leulier, Patricia Gibert, Natacha Kremer

**Affiliations:** ^1^ Université de Lyon Université Lyon 1 CNRS Laboratoire de Biométrie et Biologie Evolutive UMR 5558 Villeurbanne France; ^2^ Institut de Génomique Fonctionnelle de Lyon (IGFL) Université de Lyon Ecole Normale Supérieure de Lyon CNRS UMR 5242 Université Claude Bernard Lyon 1 Lyon France

**Keywords:** *Drosophila melanogaster*, field samples, fruit fly, larvae, PCR‐RFLP, taxonomic identification

## Abstract

The fruit fly *Drosophila melanogaster* is a model organism to study several aspects of metazoan biology. Most of the work has been conducted in adult fruit flies, including laboratory and field‐derived specimens, but *Drosophila melanogaster* larvae recently became a valuable model to better understand animal physiology, development, or host–microbe interactions. While adult flies can be easily assigned to a given *Drosophila* species based on morphological characteristics, such visual identification is more intricate at the larval stage. This could explain the limited number of studies focusing on larvae, especially field‐derived samples. Here, we developed a polymerase chain reaction‐restriction fragment length polymorphism (PCR‐RFLP) assay that discriminates *D. melanogaster* from other ecologically relevant *Drosophila* species at the larval stage. The method, which targets the *cytochrome oxidase I* (*COI*) gene, was validated using laboratory‐derived larvae from seven *D. melanogaster* populations originating from different geographic areas as well as six *Drosophila* species. We further validated this PCR‐RFLP assay in a natural context, by identifying wild larvae collected in two locations in France. Notably, among all PCR‐RFLP profiles that matched the *D. melanogaster* species, 100% were correctly identified, as confirmed by *COI* sequencing. In summary, our work provides a rapid, simple, and accurate molecular tool to identify *D. melanogaster* from field‐collected larvae.

## 1 INTRODUCTION

The fruit fly *Drosophila melanogaster* is widely used as a model organism due to its easy rearing, its genetic tractability, and several aspects of its biology that are relevant to human (Baker & Thummel, 2007; Lemaitre & Hoffmann, 2007). For instance, well‐characterized, inbred laboratory populations were extensively used to control for host genotype. Numerous studies also focused on field‐derived populations with preserved genetic diversity to address key issues in research fields such as host–microbe interactions or environment adaptation (Chandler, Lang, Bhatnagar, Eisen, & Kopp, 2011; Hoffmann & Willi, 2008).

**Figure 1:**
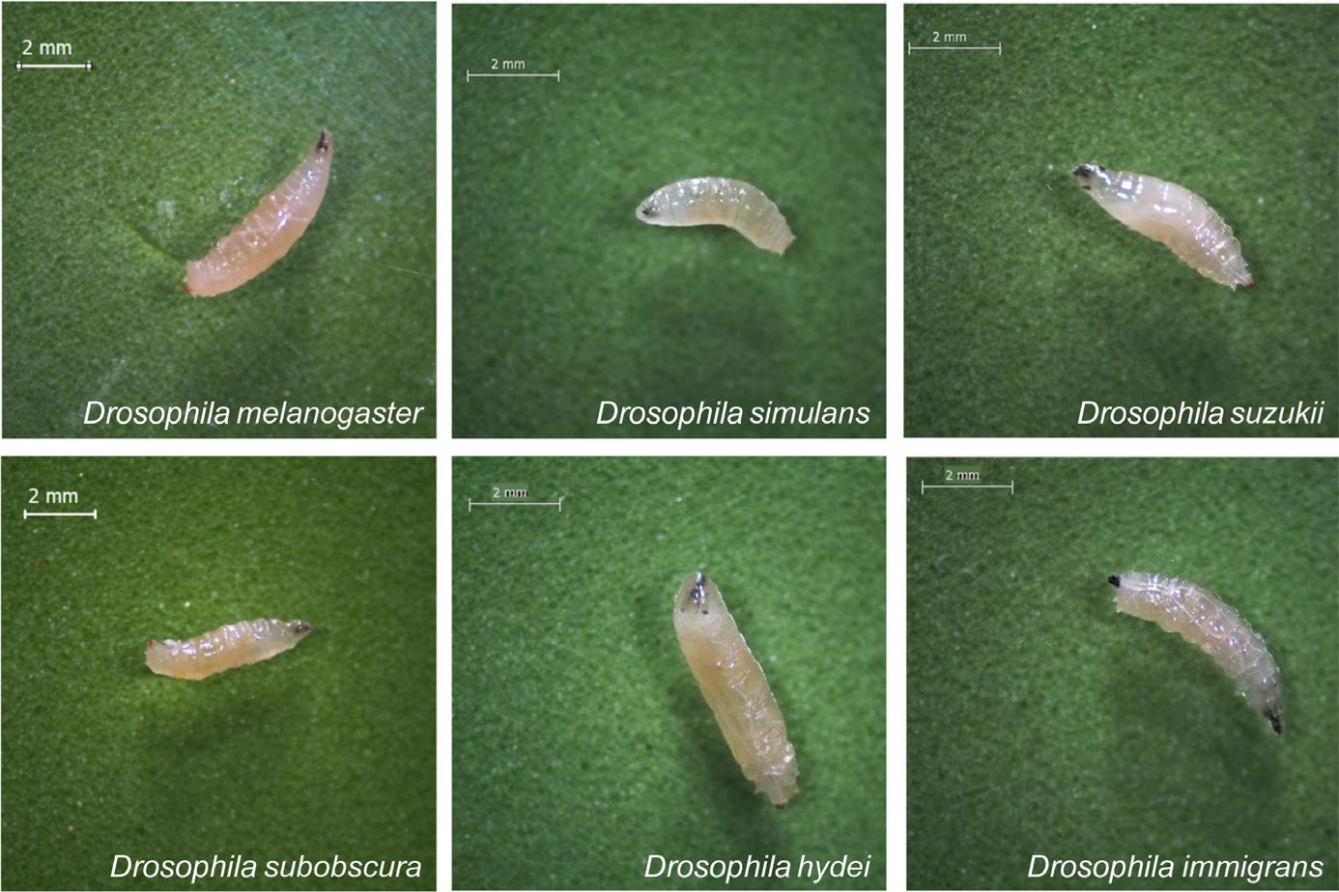
Larvae from various *Drosophila* species. Third‐instar larvae were collected as they climbed up to the tube prior to pupation. Mouth hooks at the anterior part and posterior spiracles are visible. Individuals were observed under a binocular at 20 ×  magnification

Determination of *Drosophila* species from wild‐caught adults is mainly based on morphological characteristics such as size, color, wing shape, or genital morphology (Markow & O'Grady, 2005). *Drosophila* juvenile stage (larval instars) recently appeared to be a relevant model to better understand physiology and host–microbe interaction (Erkosar et al., 2015; Storelli et al., 2011), but very few studies focussed on wild *Drosophila* larvae. In studies that did have this focus, researchers investigated larvae originating from wild‐caught adults or relied on adult emergence from field‐collected larvae to provide taxonomic identification (Durisko, Kemp, Mubasher, & Dukas, 2014; Godoy‐Herrera & Connolly, 2007; Pino et al., 2015). Wild fruit fly larvae can be distinguished at the family level—such as *Drosophilidae* and *Tephritidae* larvae—based on size or spiracles arrangement, but such visual distinction is more intricate between drosophilid species (Figure 1; Van Timmeren, Diepenbrock, Bertone, Burrack, & Isaacs, 2017). Indeed, such morphological details are not available for all the species, remain difficult to see unless performing a time‐consuming observation of all the larvae, and could vary according to environmental conditions. In this context, we developed a molecular tool that allows a rapid and accurate identification of *D. melanogaster* species at the larval stage.

We used a polymerase chain reaction‐restriction fragment length polymorphism (PCR‐RFLP) assay (Jeffreys, 1979) to identify *Drosophila* species at the larval stage. PCR‐RFLP is a rapid, affordable, and accurate tool, which was successfully applied for insect species identification, notably using mitochondrial DNA markers as a target (Kim, Tripodi, Johnson, & Szalanski, 2014; Salazar et al., 2002; Taylor, Szalanski, & Peterson, 1996). We selected the mitochondrial gene *cytochrome C oxidase I* (*COI*), as a large number of nucleotide sequences were available in public and laboratory repositories. For PCR‐RFLP development, *COI* DNA sequences from several *Drosophila* species were digested in silico using a panel of commercially available restriction enzymes. We paid specific attention to discriminate *Drosophila* species that are frequently collected along with *D. melanogaster* in French stations monitored during fieldwork (Fleury, Gibert, Ris, & Allemand, 2009). In the end, we selected the enzyme *Mbo*II, as it provided a profile specific of *D. melanogaster* among a panel of ecologically related *Drosophila* species including *D. immigrans*,* D. suzukii*,* D. subobscura*,* D. simulans*,* D. busckii,* and *D. hydei*. The method was then applied in vivo on DNA isolated from laboratory‐derived and field‐collected larvae. We validated our method by *COI* sequencing and confirmed the accuracy of species assignment using our PCR‐RFLP assay.

## 2 MATERIAL AND METHODS

### 2.1 *Drosophila* rearing and maintenance

Laboratory populations of different species from the *Drosophila* genus (*Diptera*:* Drosophilidae*) were maintained at 21°C with a 12 hr light‐dark cycle on a diet composed of 73.3 g of cornmeal (Moulin‐Giraud, France), 76.6 g of dry inactivated yeast (Lynside), 8.8 g of agar (VWR chemicals), 55.5 ml of 96% ethanol, and distilled water up to 1 L. One exception was *Drosophila suzukii* flies, which were allowed to lay eggs for 2 days on Nutri‐Fly^™^ medium (Genesee Scientific) prior transfer of the eggs on standard diet for maintenance.

### 2.2 Wild larvae collection

The two collection sites were located in organic orchards from the Rhône Valley, France: Igé (46° 23′ 56′′ N, 4° 44′ 34′′ E) and Reyrieux (45° 56′ 6′′ N, 4° 51′ 18′′ E). These sites are classically used for fieldwork in the laboratory and were chosen based on previous knowledge of autochthonous *Drosophila* species (P. Gibert, personal communication). Fieldwork was done during 2 weeks in July 2017. The day prior to depositing the traps, fresh organic fruits (*La Vie Claire*, organic market in Lyon) were bought to limit insecticide levels. The fruit baits were lacerated using a sterile scalpel to promote rotting and then placed in a perforated plastic container. Two‐thirds of banana and ~10 cherries were placed in each container on a thin layer of sawdust to keep humidity. Traps were closed and kept in a sealed box overnight to prevent any contamination by *Drosophila* from the environment. The next day, traps were humidified with tap water and suspended to the low branches of fruit trees using an iron wire, away from direct sunlight to avoid drying. Traps remained on the field for 3 days. On the third day, traps were collected and insects present inside were removed. The traps were closed with a lid and stored in the laboratory at ~25°C. Late third‐instar larvae were collected when they escaped the fruit bait prior to pupariation. All the larvae were collected using clean forceps disinfected in 70% ethanol. Larvae were rinsed for 2 min in 2.6% bleach followed by 2 min in 70% ethanol and 2 min in sterile, 1 ×  PBS to limit external DNA contamination. Larvae were observed under a stereomicroscope, and only *Drosophila*‐like larvae were selected for species identification. The selection criteria were as follows: (a) the absence of thoracic legs distinctive of *Diptera* larvae and (b) the absence of body pigmentation combined with the presence of visible branched anterior spiracles and posterior spiracles with dark orange ring at their tip, which are indicative of drosophilid, late third‐instar larvae. Larval guts were dissected and kept at −80°C for further analysis. Total genomic DNA was isolated from corresponding individual carcasses (*i.e.,* what remains after gut removal) prior to PCR‐RFLP.

### 2.3 Genomic DNA isolation

Total genomic DNA was isolated from whole individual larvae (for laboratory larvae) or individual carcasses (for wild larvae) using the 96‐well plate Animal DNA Mini‐Preps Kit (Biobasic, NBS Biologicals) according to the manufacturer's instructions with some modifications described below. Briefly, frozen samples were ground dry using a TissueLyzer (Qiagen) with one sterile 5‐mm stainless steel bead per tube for 20 s at 20 Hz. A second grinding step was performed after adding 300 μl of ACL lysis buffer and 20 μl of 20 mg/ml proteinase K per sample. Samples were then incubated overnight at 56°C. After purification according to the manufacturer's recommendations, DNA was eluted in 100 μl of DNase‐free water (Gibco). An empty tube and a tube with a grinding bead alone were included as controls to monitor DNA cross‐contamination between samples. DNA was stored at −20°C until use.

### 2.4 In silico design of the PCR‐RFLP assay

In silico design of PCR‐RFLP was performed using CLC Bio main workbench software (version 7.9.1). *Cytochrome C oxidase subunit I* (*COI*) nucleotide sequences were downloaded from the National Center for Biotechnology Information (NCBI) and aligned using multiple sequence alignment (*MUSCLE*) tool with default parameters (Edgar, 2004). We included *COI* sequences from ecologically relevant, *Drosophila*‐like species. We hypothesized that “contaminant” larvae (*i.e.,* hardly distinguishable from *D. melanogaster* in the field) would likely belong to the *Drosophilidae* family. Therefore, we included *COI* sequences from *Drosophilidae* detected in the Rhône‐Alpes region according to previous field surveys, although most of these species were sporadically observed (Withers & Allemand, 2012). We also included *COI* sequences from all the *Drosophila* species, notably the six species regularly detected in close association with *D. melanogaster* in the two selected field stations (*Drosophila immigrans, Drosophila suzukii*,* Drosophila subobscura*,* Drosophila simulans*,* Drosophila busckii,* and *Drosophila hydei*). Together, we obtained 465 unique *COI* sequences from 214 species, including 15 *Drosophilidae* species detected during field survey in Rhône‐Alpes and 199 *Drosophila* species (Supporting information Table S1). Sequences under 650 base pairs (bp) were filtered out, and only unique sequences (*i.e*., no duplicate) were retained for each species. These unique sequences were verified manually using the Basic Local Alignment Tool (BLAST) to ensure that they encode for *COI*. In silico digestion of the clean *COI* sequences was performed using the 1,562 commercially available restriction enzymes present in CLC Bio, which correspond to 340 distinct cutting sites. This method allowed us to attribute a PCR‐RFLP profile number to each species.

### 2.5 PCR‐RFLP on individual larvae

DNA samples were diluted 1:50 in DNase‐free water prior to PCR amplification, to limit potential PCR inhibition by molecules from larval extract. A 709‐bp fragment of the *cytochrome C oxidase subunit I* (*COI*) encoding gene was amplified using HCO (5′‐GGT‐CAA‐CAA‐ATC‐ATA‐AAG‐ATA‐TTG‐G‐3′) and LCO (5′‐TAA‐ACT‐TCA‐CGG‐TGA‐CCA‐AAA‐AAT‐CA‐3′) primers. The 25‐μl reaction contained 2.5 units of DreamTaq DNA polymerase (Thermo Fisher), 2.5 μl of 10× DreamTaq DNA Green Buffer, 200 μM of each dNTP (Thermo Fisher), 200 nM of each primer (Eurogentec), and 1.5 μl of diluted template DNA. PCR was performed on a Tetrad 2 Thermal cycler (Bio‐Rad) as follows: 95°C for 3 min; 40 cycles of 94°C for 45 s, 47°C for 45 s, and 72°C for 45 s; and a final extension step at 72°C for 10 min. The length of the PCR products was examined by electrophoresis on a 1.5% agarose gel under UV using a 100‐bp DNA ladder (Fermentas). An aliquot of the PCR product was used to perform restriction fragment length polymorphism (RFLP) using the *MboII* restriction enzyme (Fermentas). The 20 μl‐digestion mixture was composed of 1 μl of *MboII* (5 U/μl), 2 μl of 10× reaction buffer, 7 μl of PCR product, and 10 μl of DNase‐free water. A control sample without the restriction enzyme (replaced by water) was used as a nondigested control. The samples were incubated at 37°C for 1 hr prior to electrophoresis on a 2% agarose gel followed by UV exposure (Gel documentation Bio‐Print, Vilber). The size of the bands was estimated using a 50‐bp ladder (Fermentas).

## 3 RESULTS

### 3.1 In silico identification of *D. melanogaster* species using *COI*‐*MboII* PCR‐RFLP assay

**Figure 2:**
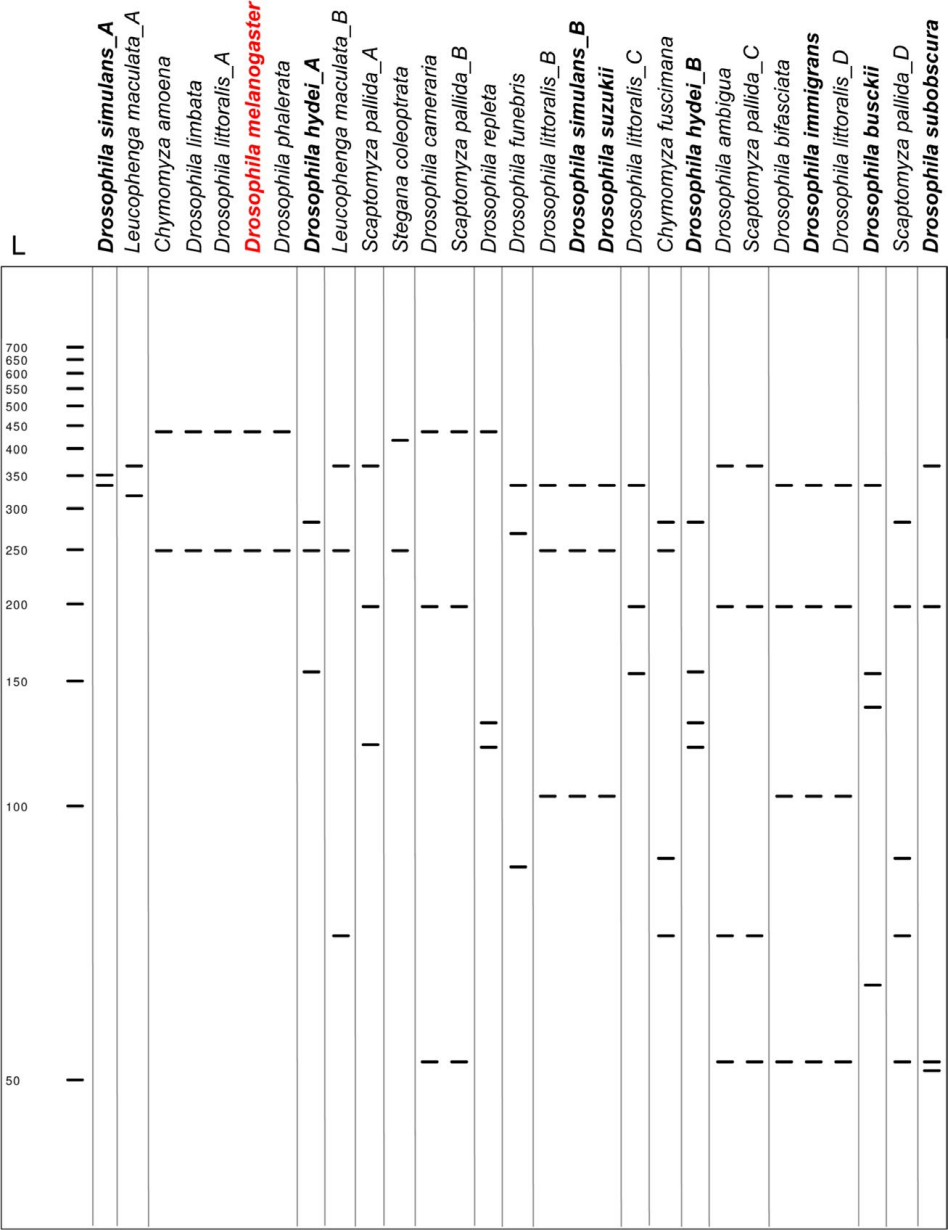
In silico PCR‐RFLP profiles of ecologically relevant *Drosophilidae* species. Nineteen unique PCR‐RFLP profiles from *Drosophilidae* species from Rhône‐Alpes region are shown following in silico digestion of a 709‐bp fragment of *COI* with *MboII* enzyme. PCR‐RFLP profile of the target species *D. melanogaster* is indicated in red. The six *Drosophila* species found in close ecological association with *D. melanogaster* in the field stations tested are indicated in bold. Each unique PCR‐RFLP profile within a species is distinguished by a letter (_A to _D). Molecular ladder (L) indicates the size of the fragments in base pairs

The selected *COI* sequences were digested in silico using the set of restriction enzymes available in CLC software (version 7.9.1). We retained commercially available restriction enzymes that display, after a single digestion step, a *D. melanogaster* profile that would be distinct from *Drosophilidae* of the region (Withers & Allemand, 2012) and especially from the co‐occurring *Drosophila* species present in the field stations. Among the potential candidate enzymes, *AciI*,* AceIII,* and *MboII* discriminated *D. melanogaster* from the *Drosophila* species detected in the two field stations. We selected *MboII* as it also provided a specific PCR‐RFLP profile for the nontarget *Drosophila* species (Supporting information Figures S1‐S2). Digestion by *MboII* provided 32 unique digestion profiles for the selected *Drosophilidae* species and 56 unique digestion profiles within the *Drosophila* genus (Supporting information Table S1). Among the 16 unique *COI* sequences of *D. melanogaster*,* MboII* digestion resulted in a single PCR‐RFLP profile. However, this profile was identical to 29 other species (Figure 2; Supporting information Table S1). To draw a workable graphical representation of this analysis, we chose to focus on the ecologically relevant species cited above (Withers & Allemand, 2012). PCR‐RFLP profiles from the 15 other members of the *Drosophilidae* family present in Rhône‐Alpes region were different from *D. melanogaster* (Figure 2). In particular, *D. melanogaster* PCR‐RFLP profile was clearly distinct from the six *Drosophila* species of ecological interest, demonstrating the accuracy of our method in this context. As justified above, the use of *MboII* digestion enzyme enabled us to specifically identify these six species, which presented a unique profile (Figure 2).

### 3.2 Species discrimination of *Drosophila* larvae in vivo using PCR‐RFLP

**Figure 3:**
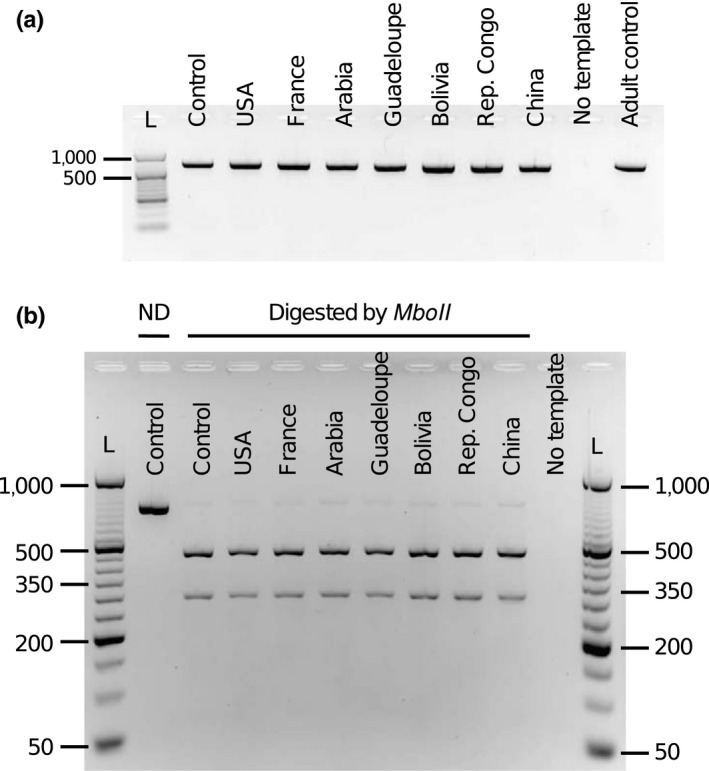
*COI* PCR‐RFLP profile is conserved among *D. melanogaster* populations. (a) PCR amplification of a 709‐bp fragment of the *COI* gene. (b) PCR‐RFLP profiles following *COI* PCR product digestion by *MboII* restriction enzyme. ND, nondigested control. Each band corresponds to a DNA sample isolated from one individual larva. DNA templates from *D. melanogaster w* ^1118^ larvae or adult were used as controls. The size in base pairs is indicated and was estimated using the 50‐bp DNA ladder (Fermentas)

Genetic divergence among populations from a given species can introduce variation in the restriction site and thus impact the accuracy of the PCR‐RFLP method. In our effort to discriminate *D. melanogaster* from other species at the larval stage, we first investigated whether different populations of *D. melanogaster* (originating from various locations worldwide) exhibited the same PCR‐RFLP profile. PCR amplification of *COI* resulted in a single fragment of 709 bp for all the populations tested (Figure 3a). For all samples, *MboII* digestion produced two fragments of ~500 and ~300 bp. The PCR‐RFLP profiles being identical across all the populations tested, the profile of *D. melanogaster* is thus particularly robust for identification (Figure 3b).

**Figure 4:**
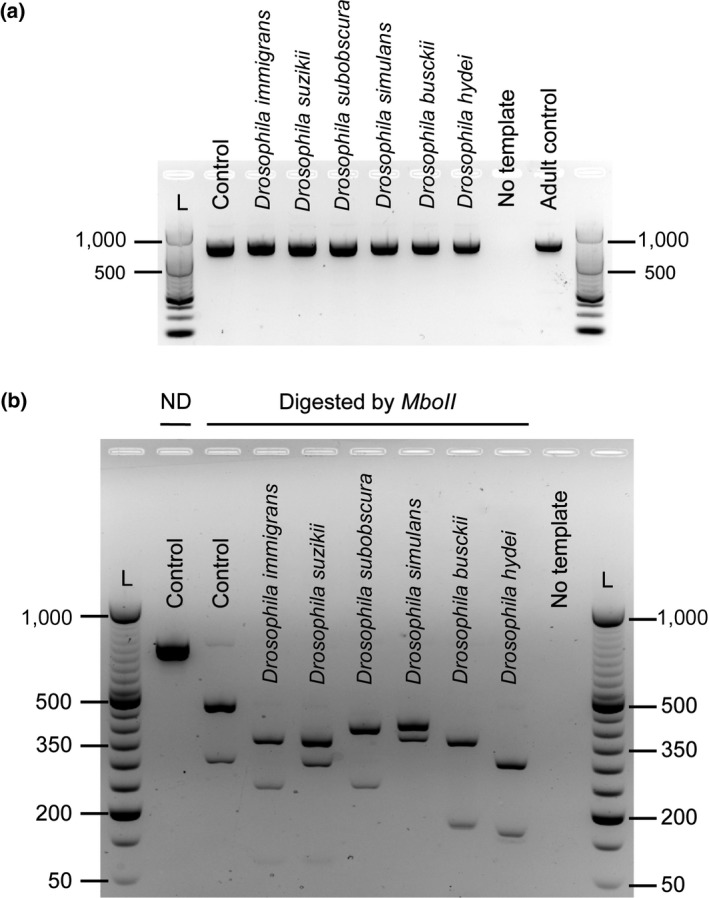
Species identification of *Drosophila* larvae using PCR‐RFLP on *COI*. (a) PCR amplification of a 709‐bp fragment of the *COI* gene. (b) PCR‐RFLP profiles following *COI* PCR product digestion by *MboII* restriction enzyme. ND, nondigested control. Each band corresponds to a DNA sample isolated from one individual larva. DNA templates from larvae or adult fly from *D. melanogaster w* ^1118^ background were used as controls. The size in base pairs is indicated according to the 50‐bp DNA ladder (Fermentas)

Then, we tested the accuracy of the in silico predictions by performing the PCR‐RFLP assay on larvae from six ecologically relevant *Drosophila* species. PCR amplification of *COI* showed a single band at the expected size of 709 bp in all the samples (Figure 4a). All the *MboII*‐digested profiles displayed between 2 and 3 bands according to the species, ranging from ~500 to ~50 bp, the band below 100 bp being hardly visible (Figure 4b). In accordance with the in silico analysis, we observed a specific PCR‐RFLP profile for *D. melanogaster*, distinguishable from the six other *Drosophila* species. Consequently, this result indicates that our method is relevant to identify *D. melanogaster* at the larval stage in this ecological context. In addition, six species‐specific profiles were obtained, suggesting that this tool could also discriminate between the selected *Drosophila* species at the larval stage (Figure 4b).

### 3.3 PCR‐RFLP implementation for species identification of wild larvae

**Figure 5:**
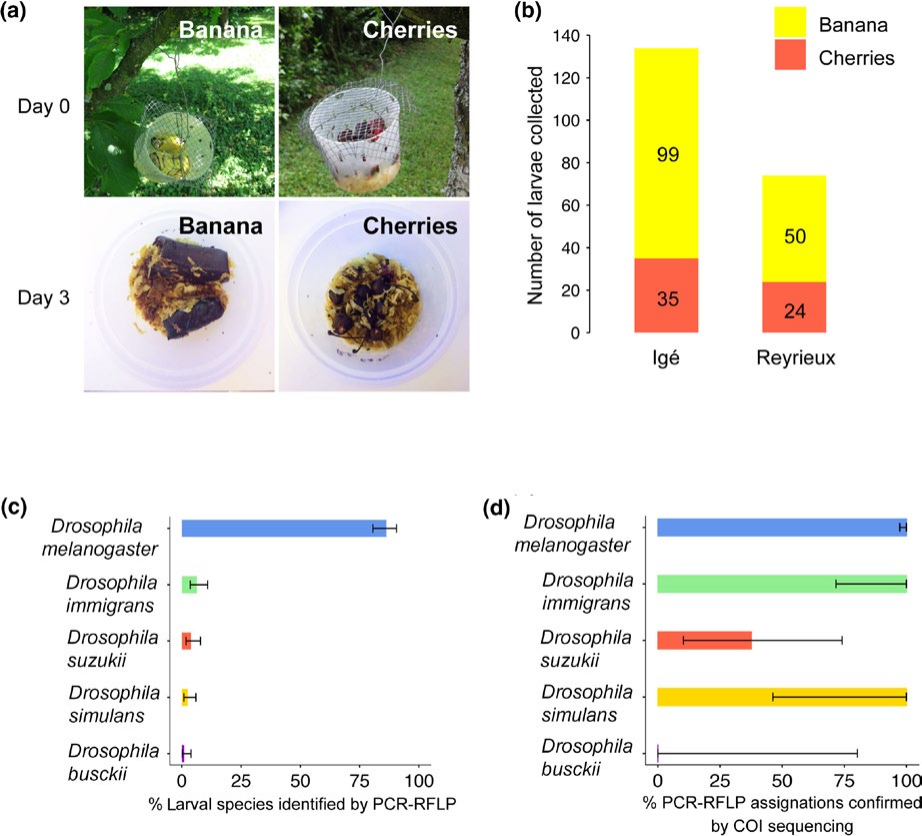
Field collection and PCR‐RFLP typing of wild *Drosophila* larvae. (a) Open traps containing fresh fruit baits (banana or cherries) at day 0 or day 3 after field deposit. (b) Number of larvae collected for each bait type in the two collection sites. (c) Proportion of each *Drosophila* species identified by PCR‐RFLP among the wild larvae collected. (d) Proportion of larvae species identified by PCR‐RFLP that was confirmed after *COI* sequencing. Bars represent 95% confidence intervals

We applied our PCR‐RFLP method to identify species from wild larvae collected in two different sites (Igé and Reyrieux) along the Rhône Valley (France) (Figure 5a‐b). Three to 15 larvae per trap were dissected and stored at −80°C prior to PCR‐RFLP analysis. From the 207 larvae collected, 86.3% (*n* = 175) presented a *D. melanogaster* PCR‐RFLP profile (Figure 5c). The remaining 13.7% could be assigned to *D. immigrans* (6.4%, *n* = 13), *D. suzukii* (3.9%, *n* = 8), *D. simulans* (2.4%, *n* = 5), or *D. busckii* (0.9%, *n* = 2), respectively (Figure 5c), with four profiles that remained unassigned due to noninterpretable profiles. To validate our visual assignment of PCR‐RFLP profiles to a given species, *COI* PCR products from all the larvae collected on the field were sequenced on the forward strand using Sanger sequencing (Biofidal). From the 175 larvae identified as *D. melanogaster* by PCR‐RFLP, 100% were confirmed to be *D. melanogaster* after *COI* sequencing (Figure 5d). The accuracy of the PCR‐RFLP method was also 100% for *D. immigrans* (*n* = 13) and *D. simulans* (*n* = 5), despite a lower number of individuals (Figure 5d). PCR‐RFLP identification of *D. busckii* and *D. suzukii* was less reliable, with 0% (0/2) and 37.5% (3/8) of the larvae species that were confirmed following *COI* sequencing, respectively (Figure 5d). The misidentified *D. suzukii* and *D. busckii* larvae belonged to a *Diptera* species of the *Drosophilidae* family, genus *Phortica* according to sequencing results. This genus was reported in France according to previous survey (Otranto, Brianti, Cantacessi, Lia, & Máca, 2006), but not in the Rhone‐Alpes region (Withers & Allemand, 2012).

## 4 DISCUSSION

PCR‐RFLP is a rapid and cost‐effective tool to determine the taxonomic rank of a biological sample, which is especially helpful in the absence of visible keys of identification. Here, we developed a PCR‐RFLP targeting the mitochondrial *COI* gene to identify *D. melanogaster* among other ecologically relevant *Drosophila* species at the larval stage.

By combining a rigorous in silico analysis with previous knowledge of the ecological context of interest, we designed an assay to discriminate *D. melanogaster* among phylogenetically close, ecologically related *Drosophila* species. As pointed out by the in silico analysis, the risk of misidentification increases with the number of species targeted. Therefore, one can optimize the PCR‐RFLP process by modifying the target gene or of the restriction enzyme, or using of a combination of several restriction enzymes. However, the accuracy of the method still relies on the number of sequences of the target gene available in public repositories and on the quality of their annotation. In this context, the choice of *COI* was relevant as a large number of *COI* sequences were available for *Drosophila*.

DNA polymorphism can occur at the population level and lead to several profiles per species. Here, similar PCR‐RFLP profiles were obtained for several *D. melanogaster* populations from very distant geographic areas. This method thus provides an interesting tool for many researchers, who are working with *D. melanogaster* worldwide, especially for the study of field‐derived samples. When we applied this method on laboratory populations, we confirmed that larvae from each of the six *Drosophila* species tested presented a unique PCR‐RFLP profile.

We finally validated our PCR‐RFLP technique using wild larvae collected on the field in two sites along the Rhône Valley, France. Around 30% of the traps were positive for larvae, suggesting that our collection method could be optimized by dissecting the fruit, or by increasing bait attractiveness using, for instance, different fruit baits or adding yeast extract. Three *Drosophila* species (*D. limbata*,* D. littoralis,* and *D. phalerata*) harbored the same in silico PCR‐RFLP profile than *D. melanogaster*. Among these species, only *D. phalerata* was known to develop on rotten fruits, and only this species was observed in the field station explored in this study (Withers & Allemand, 2012). Only 3.4% (7/207) of the collected larvae were misidentified by our PCR‐RFLP method, and the misrecognition concerned the sole *D. suzukii* and *D. busckii* species. Although high, accuracy of our assay could thus be improved on the particular “contaminant” genus/species (here *Phortica*), for instance, by performing a second digestion on this subset of samples. Further in silico analysis suggests that the enzyme *FspBI* could help to distinguish *D. melanogaster* from *C. amoena*,* D. limbata*,* D. littoralis,* and *D. phalerata* (Supporting information Figure S3). Consequently, this enzyme could be used on *COI* amplicons in a second digestion step, if the presence of flies from by these four species is suspected in the area of study.

In summary, the PCR‐RFLP is very robust and reliable for *D. melanogaster* identification, as all the 175 wild‐caught larvae assigned by PCR‐RFLP were confirmed following *COI* sequencing. This method allows a gain in time and money by eliminating the need of *COI* sequencing. This PCR‐RFLP tool was initially designed to specifically identify *D. melanogaster* in a given ecological context, and it appeared to be also robust for the identification of *D. immigrans* and *D. simulans* larvae, even if the sample size should be increased to confirm this result. More generally, the pipeline of the method can be easily adapted to identify *Drosophila* in other ecological contexts or to target other species in view of the availability of target gene sequences.

## Conflict of Interest

None declared.

## AUTHOR CONTRIBUTIONS

VR, FL, and NK designed the work. VR, PG, and NK collected field samples. HH designed the PCR‐RFLP assay. VR, NK, HH, and MV performed the experiments. VR analyzed the results and drafted the manuscript. NK and FL provided critical reading and improved the article.

## DATA ACCESSIBILITY

DNA sequences of *COI* PCR fragments from wild *Drosophila* larvae are publicly available on Dryad at https://doi.org/10.5061/dryad.pm71k02.
