# Supplementary material for: Development of a PCR‐RFLP assay to identify Drosophila melanogaster among field‐collected larvae
Source: Ecol Evol. 2018 Sep 25;8(20):10067–74. doi: 10.1002/ece3.4453 (PMC6206224; doi:10.1002/ece3.4453)
Supplement: Supplementary file 4 [file ECE3-8-10067-s004.docx]

| ***Drosophilidae* species** | **Nb. *COI* sequences** | **Nb.**  **Fragments** | **Profile**  **nb.** | **Detected in**  **Rhône-Alpes region** |
| --- | --- | --- | --- | --- |
| *Chymomyza amoena* | 1 | 2 | 4 | no |
| *Chymomyza fuscimana* | 1 | 4 | 28 | yes |
| *Drosophila affinis* | 9 | 4 | 46 | no |
| *Drosophila albomicans* | 1 | 4 | 27 | no |
| *Drosophila allochroa* | 1 | 4 | 29 | no |
| *Drosophila americana* | 1 | 4 | 49 | no |
| *Drosophila amplipennis* | 1 | 5 | 63 | no |
| *Drosophila ananassae* | 1 | 3 | 24 | no |
| *Drosophila anceps* | 1 | 5 | 51 | no |
| *Drosophila angor* | 1 | 1 | 0 | no |
| *Drosophila angor* | 1 | 4 | 36 | no |
| *Drosophila angor* | 1 | 4 | 42 | no |
| *Drosophila angor* | 1 | 4 | 48 | no |
| *Drosophila angor* | 4 | 3 | 7 | no |
| *Drosophila antonietae* | 1 | 3 | 14 | no |
| *Drosophila aracataca* | 1 | 4 | 32 | no |
| *Drosophila arawakana* | 1 | 2 | 4 | no |
| *Drosophila arizonae* | 1 | 3 | 14 | no |
| *Drosophila asahinai* | 2 | 1 | 0 | no |
| *Drosophila asper* | 1 | 5 | 62 | no |
| *Drosophila auraria* | 1 | 3 | 22 | no |
| *Drosophila bai* | 1 | 3 | 19 | no |
| *Drosophila baimaii* | 1 | 4 | 46 | no |
| *Drosophila bakoue* | 1 | 2 | 2 | no |
| *Drosophila barbarae* | 1 | 2 | 4 | no |
| *Drosophila barbarae* | 2 | 2 | 2 | no |
| *Drosophila barutani* | 1 | 4 | 35 | no |
| *Drosophila barutani* | 1 | 5 | 56 | no |
| *Drosophila beppui* | 1 | 4 | 49 | no |
| *Drosophila beppui* | 2 | 3 | 25 | no |
| *Drosophila biauraria* | 2 | 3 | 22 | no |
| *Drosophila bicornuta* | 2 | 2 | 2 | no |
| *Drosophila bipatita* | 1 | 3 | 24 | no |
| *Drosophila birchii* | 1 | 2 | 2 | no |
| *Drosophila biseriata* | 1 | 2 | 2 | no |
| *Drosophila bocki* | 1 | 3 | 22 | no |
| *Drosophila bocqueti* | 1 | 2 | 2 | no |
| *Drosophila borusta* | 1 | 2 | 4 | no |
| *Drosophila brachytarsa* | 1 | 5 | 64 | no |
| *Drosophila bromeliae* | 1 | 3 | 24 | no |
| *Drosophila bunnanda* | 1 | 3 | 22 | no |
| *Drosophila burlai* | 1 | 2 | 2 | no |
| *Drosophila buzzatii* | 1 | 3 | 14 | no |
| *Drosophila canalinea* | 1 | 3 | 18 | no |
| *Drosophila capricorni* | 1 | 3 | 24 | no |
| *Drosophila cardini* | 1 | 1 | 0 | no |
| *Drosophila cardinoides* | 1 | 2 | 4 | no |
| *Drosophila caribiana* | 1 | 2 | 4 | no |
| *Drosophila cauverii* | 1 | 2 | 2 | no |
| *Drosophila cestri* | 1 | 4 | 31 | no |
| *Drosophila chauvacae* | 1 | 2 | 2 | no |
| *Drosophila clefta* | 1 | 3 | 19 | no |
| *Drosophila comatifemora* | 1 | 3 | 15 | no |
| *Drosophila conformis* | 1 | 4 | 41 | no |
| *Drosophila cordeiroi* | 1 | 3 | 7 | no |
| *Drosophila daruma* | 4 | 5 | 62 | no |
| *Drosophila dentissima* | 1 | 3 | 24 | no |
| *Drosophila dentissima* | 1 | 6 | 69 | no |
| *Drosophila diplacantha* | 1 | 2 | 2 | no |
| *Drosophila dunni* | 1 | 2 | 4 | no |
| *Drosophila dyaramankana* | 1 | 4 | 39 | no |
| *Drosophila emarginata* | 1 | 3 | 24 | no |
| *Drosophila erecta* | 1 | 3 | 14 | no |
| *Drosophila eurypeza* | 1 | 5 | 57 | no |
| *Drosophila ezoana* | 1 | 2 | 5 | no |
| *Drosophila falleni* | 1 | 3 | 18 | no |
| *Drosophila fengkainensis* | 2 | 2 | 2 | no |
| *Drosophila flavopilosa* | 1 | 3 | 24 | no |
| *Drosophila fluvialis* | 1 | 6 | 68 | no |
| *Drosophila fraburu* | 1 | 3 | 8 | no |
| *Drosophila gani* | 1 | 5 | 67 | no |
| *Drosophila greeni* | 1 | 3 | 11 | no |
| *Drosophila griseolineata* | 1 | 2 | 4 | no |
| *Drosophila haleakalae* | 1 | 4 | 39 | no |
| *Drosophila hei* | 1 | 2 | 1 | no |
| *Drosophila hirtipes* | 1 | 4 | 44 | no |
| *Drosophila huaylasi* | 1 | 2 | 4 | no |
| *Drosophila hypocausta* | 1 | 6 | 70 | no |
| *Drosophila imparisetae* | 1 | 4 | 41 | no |
| *Drosophila inca* | 1 | 3 | 14 | no |
| *Drosophila incompta* | 1 | 1 | 0 | no |
| *Drosophila incompta* | 2 | 2 | 4 | no |
| *Drosophila innubila* | 19 | 3 | 18 | no |
| *Drosophila jambulina* | 1 | 2 | 2 | no |
| *Drosophila jambulina* | 2 | 1 | 0 | no |
| *Drosophila kambysellisi* | 1 | 3 | 15 | no |
| *Drosophila kanapiae* | 1 | 4 | 46 | no |
| *Drosophila kanekoi* | 1 | 3 | 24 | no |
| *Drosophila karakasa* | 1 | 5 | 66 | no |
| *Drosophila kepulauana* | 1 | 4 | 27 | no |
| *Drosophila kikkawai* | 1 | 3 | 22 | no |
| *Drosophila kohkoa* | 1 | 3 | 10 | no |
| *Drosophila lacertosa* | 1 | 3 | 25 | no |
| *Drosophila lacicola* | 1 | 4 | 49 | no |
| *Drosophila lacteicornis* | 1 | 1 | 0 | no |
| *Drosophila lamottei* | 1 | 4 | 31 | no |
| *Drosophila latifshahi* | 1 | 4 | 44 | no |
| *Drosophila leontia* | 1 | 3 | 22 | no |
| *Drosophila lini* | 2 | 3 | 22 | no |
| *Drosophila longiserrata* | 1 | 4 | 39 | no |
| *Drosophila lummei* | 1 | 3 | 25 | no |
| *Drosophila machalilla* | 1 | 2 | 4 | no |
| *Drosophila macroptera* | 1 | 3 | 18 | no |
| *Drosophila malagassya* | 2 | 3 | 11 | no |
| *Drosophila malerkotliana* | 1 | 3 | 25 | no |
| *Drosophila matilei* | 1 | 3 | 24 | no |
| *Drosophila mauritiana* | 1 | 3 | 24 | no |
| *Drosophila mayri* | 1 | 2 | 2 | no |
| *Drosophila mayri* | 1 | 3 | 22 | no |
| *Drosophila medioconstricta* | 1 | 3 | 19 | no |
| *Drosophila mediostriata* | 1 | 2 | 4 | no |
| *Drosophila megapyga* | 1 | 2 | 2 | no |
| *Drosophila melanica* | 1 | 3 | 19 | no |
| *Drosophila mercatorum* | 1 | 3 | 17 | no |
| *Drosophila mettleri* | 1 | 2 | 2 | no |
| *Drosophila micromettleri* | 1 | 1 | 0 | no |
| *Drosophila microralis* | 1 | 5 | 63 | no |
| *Drosophila mimica* | 1 | 3 | 15 | no |
| *Drosophila mojavensis* | 1 | 3 | 14 | no |
| *Drosophila montana* | 1 | 5 | 58 | no |
| *Drosophila montium* | 1 | 2 | 2 | no |
| *Drosophila multidentata* | 1 | 5 | 66 | no |
| *Drosophila munda* | 1 | 2 | 4 | no |
| *Drosophila nannoptera* | 1 | 4 | 48 | no |
| *Drosophila nasuta* | 1 | 4 | 27 | no |
| *Drosophila navojoa* | 1 | 3 | 14 | no |
| *Drosophila nebulosa* | 1 | 2 | 4 | no |
| *Drosophila neoasahinai* | 1 | 1 | 0 | no |
| *Drosophila neocardini* | 1 | 2 | 4 | no |
| *Drosophila neohypocausta* | 1 | 4 | 27 | no |
| *Drosophila neokadai* | 1 | 4 | 46 | no |
| *Drosophila neomorpha* | 1 | 2 | 4 | no |
| *Drosophila neotestacea* | 47 | 3 | 12 | no |
| *Drosophila nigella* | 1 | 5 | 64 | no |
| *Drosophila nigra* | 1 | 4 | 49 | no |
| *Drosophila nigrodunni* | 1 | 2 | 4 | no |
| *Drosophila nigrospiracula* | 1 | 3 | 9 | no |
| *Drosophila nikananu* | 1 | 3 | 22 | no |
| *Drosophila nitida* | 1 | 4 | 46 | no |
| *Drosophila novamexicana* | 1 | 4 | 49 | no |
| *Drosophila nutrita* | 1 | 5 | 64 | no |
| *Drosophila ogumai* | 1 | 3 | 22 | no |
| *Drosophila ohnishii* | 2 | 3 | 22 | no |
| *Drosophila orena* | 1 | 4 | 46 | no |
| *Drosophila ornatipennis* | 1 | 3 | 7 | no |
| *Drosophila orosa* | 1 | 2 | 2 | no |
| *Drosophila pachea* | 1 | 1 | 0 | no |
| *Drosophila pachea* | 1 | 2 | 4 | no |
| *Drosophila palustris* | 1 | 1 | 0 | no |
| *Drosophila parabipectinata* | 1 | 3 | 25 | no |
| *Drosophila paramelanica* | 1 | 3 | 19 | no |
| *Drosophila parthenogenetica* | 1 | 2 | 4 | no |
| *Drosophila parvula* | 1 | 3 | 14 | no |
| *Drosophila paulistorum* | 1 | 3 | 7 | no |
| *Drosophila pavani* | 1 | 3 | 7 | no |
| *Drosophila pectinifera* | 1 | 1 | 0 | no |
| *Drosophila pectinitarsus* | 1 | 4 | 41 | no |
| *Drosophila percnosoma* | 1 | 3 | 19 | no |
| *Drosophila perlucida* | 1 | 6 | 72 | no |
| *Drosophila pilosa* | 1 | 6 | 72 | no |
| *Drosophila polychaeta* | 1 | 4 | 43 | no |
| *Drosophila polychaeta* | 1 | 5 | 62 | no |
| *Drosophila polymorpha* | 1 | 1 | 0 | no |
| *Drosophila potamophila* | 1 | 4 | 37 | no |
| *Drosophila procardinoides* | 1 | 2 | 4 | no |
| *Drosophila pseudoananassae* | 1 | 3 | 25 | no |
| *Drosophila pseudobaimaii* | 1 | 3 | 22 | no |
| *Drosophila pseudoobscura* | 1 | 5 | 65 | no |
| *Drosophila pseudotalamancana* | 1 | 5 | 64 | no |
| *Drosophila pulaua* | 1 | 3 | 10 | no |
| *Drosophila punctatonervosa* | 1 | 2 | 5 | no |
| *Drosophila punjabiensis* | 1 | 2 | 2 | no |
| *Drosophila putrida* | 1 | 2 | 4 | no |
| *Drosophila quadraria* | 1 | 3 | 22 | no |
| *Drosophila quadrisetata* | 1 | 6 | 72 | no |
| *Drosophila quinaria* | 1 | 3 | 24 | no |
| *Drosophila quinaria* | 9 | 2 | 4 | no |
| *Drosophila recens* | 66 | 2 | 4 | no |
| *Drosophila rellima* | 1 | 3 | 24 | no |
| *Drosophila robusta* | 1 | 3 | 19 | no |
| *Drosophila rubida* | 1 | 6 | 70 | no |
| *Drosophila rufa* | 1 | 1 | 0 | no |
| *Drosophila santomea* | 1 | 5 | 63 | no |
| *Drosophila sechellia* | 1 | 3 | 24 | no |
| *Drosophila seguyi* | 1 | 1 | 0 | no |
| *Drosophila seguyi* | 1 | 2 | 2 | no |
| *Drosophila seguyi* | 1 | 3 | 11 | no |
| *Drosophila serrata* | 2 | 3 | 22 | no |
| *Drosophila silvestris* | 1 | 3 | 15 | no |
| *Drosophila soonae* | 1 | 4 | 47 | no |
| *Drosophila subauraria* | 1 | 3 | 22 | no |
| *Drosophila subpulchrella* | 1 | 3 | 24 | no |
| *Drosophila subquinaria* | 2 | 2 | 4 | no |
| *Drosophila sulfurigaster* | 1 | 4 | 27 | no |
| *Drosophila tani* | 1 | 2 | 2 | no |
| *Drosophila teissieri* | 1 | 5 | 63 | no |
| *Drosophila trapezifrons* | 1 | 3 | 15 | no |
| *Drosophila triauraria* | 1 | 3 | 22 | no |
| *Drosophila tripunctata* | 1 | 2 | 3 | no |
| *Drosophila truncata* | 1 | 2 | 2 | no |
| *Drosophila tsacasi* | 1 | 2 | 2 | no |
| *Drosophila tsigana* | 2 | 4 | 39 | no |
| *Drosophila tsukubaensis* | 1 | 5 | 55 | no |
| *Drosophila velox* | 1 | 4 | 45 | no |
| *Drosophila virilis* | 1 | 5 | 66 | no |
| *Drosophila vulcana* | 1 | 3 | 22 | no |
| *Drosophila wassermani* | 1 | 3 | 23 | no |
| *Drosophila watanabei* | 1 | 2 | 2 | no |
| *Drosophila willistoni* | 12 | 3 | 7 | no |
| *Drosophila yakuba* | 5 | 5 | 63 | no |
| *Drosophila yunnanensis* | 1 | 3 | 26 | no |
| *Drosophila acanthomera* | 1 | 3 | 19 | no |
| *Drosophila acanthoptera* | 1 | 2 | 4 | no |
| *Drosophila acutilabella* | 1 | 2 | 4 | no |
| *Drosophila adamsi* | 2 | 4 | 44 | no |
| *Drosophila adamsi* | 2 | 5 | 53 | no |
| *Drosophila ambigua* | 1 | 4 | 39 | yes |
| *Drosophila bifasciata* | 1 | 4 | 49 | yes |
| ***Drosophila busckii*** | 4 | 4 | 50 | yes |
| *Drosophila cameraria* | 1 | 3 | 19 | yes |
| *Drosophila funebris* | 1 | 3 | 23 | yes |
| ***Drosophila hydei*** | 2 | 3 | 7 | yes |
| ***Drosophila hydei*** | 2 | 4 | 30 | yes |
| ***Drosophila immigrans*** | 5 | 4 | 49 | yes |
| ***Drosophila immigrans*** | 5 | 4 | 49 | yes |
| *Drosophila limbata* | 1 | 2 | 4 | yes |
| *Drosophila littoralis* | 1 | 2 | 4 | yes |
| *Drosophila littoralis* | 1 | 3 | 24 | yes |
| *Drosophila littoralis* | 1 | 4 | 49 | yes |
| *Drosophila littoralis* | 3 | 3 | 25 | yes |
| ***Drosophila melanogaster*** | **16** | 2 | 4 | yes |
| *Drosophila phalerata* | 1 | 2 | 4 | yes |
| *Drosophila repleta* | 1 | 3 | 21 | yes |
| ***Drosophila simulans*** | 2 | 3 | 24 | yes |
| ***Drosophila simulans*** | 4 | 2 | 1 | yes |
| ***Drosophila subobscura*** | 1 | 5 | 59 | yes |
| ***Drosophila suzukii*** | 4 | 3 | 24 | yes |
| *Leucophenga maculata* | 1 | 3 | 14 | yes |
| *Leucophenga maculata* | 1 | 2 | 2 | yes |
| *Stegana coleoptrata* | 1 | 3 | 18 | yes |
| *Scaptomyza pallida* | 1 | 4 | 39 | yes |
| *Scaptomyza pallida* | 1 | 4 | 50 | yes |
| *Scaptomyza pallida* | 1 | 3 | 19 | yes |
| *Scaptomyza pallida* | 1 | 3 | 15 | yes |
| *Scaptomyza pallida* | 1 | 5 | 52 | yes |
